# Post-extubation dysphagia is associated with longer hospitalization in survivors of critical illness with neurologic impairment

**DOI:** 10.1186/cc12791

**Published:** 2013-06-20

**Authors:** Madison Macht, Christopher J King, Tim Wimbish, Brendan J Clark, Alexander B Benson, Ellen L Burnham, André Williams, Marc Moss

**Affiliations:** 1 Division of Pulmonary Sciences and Critical Care Medicine, University of Colorado Denver, 12700 E 19th Avenue, Aurora, CO 80045, USA; 2 University of Colorado Hospital, Rehabilitation Therapy, 12700 E 19th Avenue, Aurora, CO 80045, USA; 3 Division of Biostatistics and Bioinformatics, National Jewish Health, 1400 Jackson Street, Denver, CO 80206, USA

**Keywords:** mechanical ventilation, intubation, intratracheal, swallowing disorders, dysphagia, aspiration, respiratory

## Abstract

**Introduction:**

Critically ill patients can develop acute respiratory failure requiring endotracheal intubation. Swallowing dysfunction after liberation from mechanical ventilation, also known as post-extubation dysphagia, is common and deleterious among patients without neurologic disease. However, the risk factors associated with the development of post-extubation dysphagia and its effect on hospital lengthofstay in critically ill patients with neurologic disorders remains relatively unexplored.

**Methods:**

We conducted a retrospective, observational cohort study from 2008 to 2010 of patients with neurologic impairment who required mechanical ventilation and subsequently received a bedside swallow evaluation (BSE) by a speech-language pathologist.

**Results:**

A BSE was performed after mechanical ventilation in 25% (630/2,484) of all patients. In the 184 patients with neurologic impairment, post-extubation dysphagia was present in 93% (171/184), and was classified as mild, moderate, or severe in 34% (62/184), 26% (48/184), and 33% (61/184), respectively. In univariate analyses, statistically significant risk factors for moderate/severe dysphagia included longer durations of mechanical ventilation and the presence of a tracheostomy. In multivariate analysis, adjusting for age, tracheostomy, cerebrovascular disease, and severity of illness, mechanical ventilation for >7 days remained independently associated with moderate/severe dysphagia (adjusted odds ratio = 4.48 (95%confidence interval = 2.14 to 9.81), *P*<0.01). The presence of moderate/severe dysphagia was also significantly associated with prolonged hospital lengthofstay, discharge status, and surgical placement of feeding tubes. When adjusting for age, severity of illness, and tracheostomy, patients with moderate/severe dysphagia stayed in the hospital 4.32 days longer after their initial BSE than patients with none/mild dysphagia (95% confidence interval = 3.04 to 5.60 days, *P *<0.01).

**Conclusion:**

In a cohort of critically ill patients with neurologic impairment, longer duration of mechanical ventilation is independently associated with post-extubation dysphagia, and the development of post-extubation dysphagia is independently associated with a longer hospital length of stay after the initial BSE.

## Introduction

Patients with both acute and pre-existing neurologic disorders can develop acute respiratory failure requiring endotracheal intubation and mechanical ventilation in a neurocritical care unit [1-3]. While intubated patients with acute cerebrovascular disease have a hospital mortality of approximately 50% [1,4,5], those with other neuromuscular diseases, such as movement disorders and peripheral neuropathy, have an estimated hospital mortality of only 15% [6]. Collectively, survivors of both cerebrovascular and other neurologic diseases who have been intubated have a median survival of over 3 years, yet suffer from eating difficulty, respiratory impairment, neurocognitive disorders, and overall decreased qualityoflife [4,7].

Increasingly, attention has focused on dysfunctional swallowing in patients recovering from acute respiratory failure and endotracheal intubation [8,9]. Also known as post-extubation dysphagia, swallowing dysfunction following intubation is the inability to effectively transfer food and liquid from the mouth into the stomach. Post-extubation dysphagia can occur in patients in a neurocritical care unit either as a result of the underlying initial neurologic disease, or due to new bulbar, sensory, or cognitive abnormalities caused by critical illnesses and/or endotracheal intubation. Dysphagia in noncritically ill patients with cerebrovascular disease has been shown to result in aspiration, pneumonia, and increased mortality [10-12]. Additionally, recent data in extubated acute respiratory failure survivors without cerebrovascular or other neuromuscular diseases show an association between more severe dysphagia and poorer patient outcomes, including pneumonia, reintubation, and mortality [8].

The existence of dysphagia in recentlyextubated critically ill patients with neuromuscular or cerebrovascular impairment, its risk factors, and the effects of this dysphagia on patient outcomes are relatively unexplored. We therefore sought to identify specific risk factors associated with dysphagia in recentlyextubated critically ill patients with neuromuscular or cerebrovascular impairment, and to define the effects of post-extubation dysphagia on their outcomes. We hypothesized that the duration of mechanical ventilation would be a risk factor for dysphagia following extubation, and that patients with more severe dysphagia would have longer hospital lengths of stay when compared with patients with mild or no dysphagia.

## Materials and methods

### Study design

Using the University of Colorado Hospital medical records system, we conducted a retrospective, observational cohort study. Patients were eligible if they met all of the following criteria: admission to an ICU during the 2-year period from April 2008 to April 2010; receipt of mechanical ventilation for any duration; presence of an acute or pre-existing neuromuscular or cerebrovascular disease resulting in ICU admission; and age ≥18 years. We included patients who received shorter (<48 hours) durations of mechanical ventilation because previous authors have suggested that short-term endotracheal intubation may result in swallowing dysfunction [13,14]. Patients were excluded if they: died prior to extubation; did not receive a bedside swallow evaluation (BSE) by a speech-language pathologist; or received their first BSE prior to initiation of mechanical ventilation. We previously reported the results of the cohort of patients free from neuromuscular or cerebrovascular disease [8]. The decision to consult a speech-language pathologist in this study was left to the discretion of the primary treating physicians. The Colorado Multiple Institutional Review Board approved both the study protocol and a waiver of informed consent.

### Data collection

Patients who received a BSE were identified in a speech-languagepathology database of completed evaluations. Patient data were obtained from admission notes, progress notes, discharge summaries, ICU flow sheets, laboratory results, primary radiologic data, and an internal diagnostic coding database. Underlying neurologic disorders were grouped into seven categories: acute hemorrhagic cerebrovascular accident (CVA); acute thrombotic CVA; CVA without further classification; peripheral neuropathy (hereditary, inflammatory, and toxic); movement disorders (including Parkinson's disease and anterior horn cell disease); multiple sclerosis (or other demyelinating diseases); and other (including neuromuscular junction diseases, myopathy, cerebral palsy, and facial nerve disorders). When multiple neurologic disorders were present for a single patient, the most dominant or limiting condition was used for this classification.

### Data analysis

Our first analysis sought to determine whether the duration of endotracheal intubation was independently associated with the presence of swallowing dysfunction in recentlyextubated patients with neuromuscular or cerebrovascular diseases. In this analysis, the primary independent variable of interest was the duration of mechanical ventilation, and secondary variables of interest included reintubation, endotracheal tube size, severity of illness, and the underlying neurologic disorder. The duration of mechanical ventilation was calculated using the hospital database. Endotracheal tube size was recorded from a respiratory therapy database and corresponded to the internal diameter of the endotracheal tube in millimeters. Severity of illness was measured using the Sequential Organ Failure Assessment (SOFA) score and was calculated at the time of admission to the ICU [15]. The ratio of the partial pressure of arterial oxygen to the fraction of inspired oxygen was corrected for the altitude and mean atmospheric pressure in Denver:

$$\text{P}/)\text{F ratio SOFA = }\left(\text{P}/)\text{F ratio Denver}\right)/)0.826$$

We omitted the component of the SOFA score corresponding to the Glasgow Coma Score as these data were not routinely available. When examining reintubation, we recorded the timing of reintubation in relation to the initial BSE. For patients with a tracheostomy, the extubation date was defined as the date of removal from positive pressure ventilation.

Our primary outcome variable for this analysis was the presence of swallowing dysfunction as determined by certified speech-language pathologists. BSEs consisted of: patient history; examination of oral, laryngeal, and vocal cord swallowing exercises; swallowing trials with different food and liquid consistencies; and assessment of swallowing function with various compensatory techniques. Speech-language pathologists classified their assessment of dysphagia as either mild, moderate, or severe based on the American Speech-Language-Hearing Association National Outcomes Measurement System Swallowing Functional Measure [16]. This is a nationallytaught, seven-point BSE scale approved by the Centers for Medicare and Medicaid Services that incorporates perceived aspiration risk and subsequent dietary recommendations. Normal swallowing was defined as an American Speech-Language-Hearing Association National Outcomes Measurement System Swallowing Functional Measure of 7, mild dysphagia was defined as a score of 5 to 6, moderate dysphagia was defined as a score of 3 to 4, and severe dysphagia was defined as a score of 1 to 2. Food and liquid consistencies used by speech-language pathologists were consistent with published diets described by the American Dietetics Association [17]. The decision to perform a videofluoroscopic swallow study (also known as a modified barium swallow) was made either by the speech-language pathologist or by the treating physician. Given our predetermined assessment of the most clinically relevant outcomes, for subsequent analyses we combined moderate and severe dysphagia into one category, and the absence of dysphagia or mild dysphagia into another category.

In our second analysis, we determined the effect of the presence of swallowing dysfunction on the duration of hospitalization following the initial BSE. Secondary outcome variables included: the need for reintubation; the development of hospital-acquired pneumonia; hospital length of stay; the surgical placement of a feeding tube; in-hospital mortality; and the composite of hospital-acquired pneumonia, reintubation, or death [8]. Our outcome variables were defined using the following criteria. Reintubation was defined as the placement of an endotracheal tube for any reason after the initial endotracheal tube had been removed. The diagnosis of hospital-acquired pneumonia required the presence of criteria as defined in the American Thoracic Society/Infectious Diseases Society of America guideline [18] as well as the decision of the treating physician to administer antimicrobial treatment. For hospital length of stay, we recorded both total hospital days as well as the duration in the hospital after the initial BSE. Feeding tube placement was defined as the surgical placement of a gastric or jejunal tube by a surgeon, gastroenterologist, or interventional radiologist.

### Statistical analysis

Data that were not normally distributed are reported as the median (25 to 75% interquartile range). Univariate comparisons were evaluated with chi-square or Kruskal-Wallis tests, as appropriate. Nonparametric tests were used when data were not normally distributed. Backwards logistic regression models were used to determine the effect of the duration of mechanical ventilation on the presence of dysphagia, and the effect of dysphagia on patient outcomes. Due to interaction between tracheostomy and the duration of mechanical ventilation in the models examining the effect of the duration of mechanical ventilation on the presence of dysphagia, we performed separate multivariate analyses for those patients with and without tracheostomy. SAS 9.1 (SAS Institute Inc., Cary, NC, USA) was used for all analyses, and *P*<0.05 was considered statistically significant. Confidence intervals (95%) for adjusted odds ratios and the 25 to 75% interquartile range for median values are recorded in parentheses.

## Results

**Figure 1:**
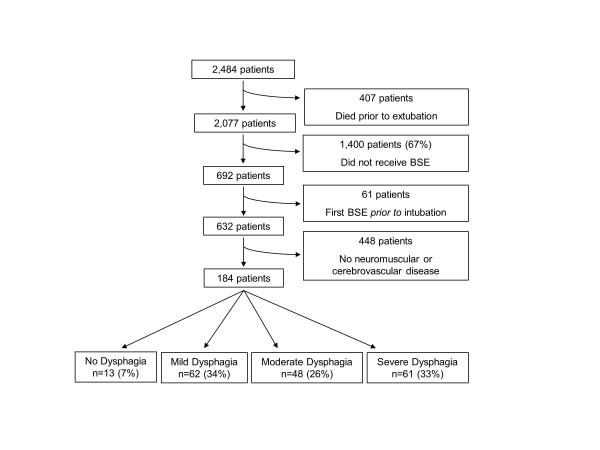
**Flowchart detailing enrollment of subjects**. BSE, bedside swallow evaluation.

Study enrollment is outlined in Figure 1. Of the 2,484 patients who met the inclusion criteria, 407 died prior to extubation. Of the remaining patients, 67% (1,400/2,077) did not receive a BSE. A physician's order to perform a BSE was most common for patients on a neurological service (45%), followed by those on a medical service (34%) and on a surgical service (17%)(*P *<0.001). Of the remaining 692 patients who received a BSE during their admission, 61 were excluded because the initial BSE had been done prior to intubation, and 448 were excluded because they did not have a diagnosis of either neuromuscular or cerebrovascular disease. The remaining 184 patients were included in our final analysis.

Of the 184 patients in the final analysis, the mean age was 55 ± 15 years and 51% were male. On average, patients received 7 days of mechanical ventilation (3 to 13 days), and stayed in the ICU for 11 days (6 to 19 days) and in the hospital for 20 days (13 to 33 days). They remained in the hospital for 10 days (5 to 17 days) after their first extubation. A total of 39% (72/184) of patients had a noncerebrovascular disease-related diagnosis. Of those without cerebrovascular disease, 26% (19/72) were diagnosed with peripheral neuropathy, 24% (17/72) had a movement disorder, 10% (7/72) had multiple sclerosis, and 40% (29/72) had another primary neurologic disease. Of the 61% (112/184) of patients with cerebrovascular disease, a total of 57% (64/112) had an acute hemorrhagic CVA, while 33% (37/112) had an acute thrombotic CVA. Twenty percent of patients (36/184) had a tracheostomy at the time of the initial BSE. In this cohort of patients who survived to be extubated, in-hospitalmortality was only 2% (4/184), although only 28% (51/184) of patients were discharged to their homes. A total of 53% (97/184) of patients were not allowed to take any food or liquids after their first BSE, and 22% (41/184) had a feeding tube placed surgically.

Dysphagia was present in 93% (171/184) of these patients. Dysphagia severity was classified as mild in 34% (62/184) of the total cohort, moderate in 26% (48/184), and severe in 33% (61/184). A total of 16 of the 184 patients (9%) received a modified barium swallow in addition to a BSE. The median time from extubation to the initial BSE was 1 day (1 to 3 days) and did not differ significantly between those with none/mild dysphagia (1 day (1 to 2 days)) and those with moderate/severe dysphagia (2 days (1 to 3.5 days), *P *=0.28).

Univariate analyses evaluating for patient characteristics associated with the presence of moderate/severe post-extubation dysphagia are described in Table 1. Patients with moderate/severe dysphagia were more likely to have had a longer duration of mechanical ventilation and a tracheostomy performed. The presence of primary cerebrovascular disease was not significantly associated with the development of moderate/severe dysphagia when compared with patients with other neuromuscular disorders. Due to a significant interaction between tracheostomy and duration of mechanical ventilation, we performed two separate multivariate analyses: one for patients with tracheostomy, and one for patients without tracheostomy. In the analysis of patients without tracheostomy, adjusting for age, cerebrovascular disease, and severity of illness, mechanical ventilation for >7 days remained independently associated with moderate/severe dysphagia (adjusted odds ratio = 4.59 (2.16 to 10.27), *P*<0.01). In the analysis of patients with tracheostomy (*n*=36), the duration of mechanical ventilation was not independently associated with moderate/severe dysphagia.

**Table 1:** Univariate analysis of risk factors for post-extubation dysphagia

	Dysphagia severity		
		
Characteristic	None/mild (*n *= 75)	Moderate/severe (*n *= 109)	*P *value
Age (years)	58 ± 14	53 ± 15	0.07
Male	37 (49)	56 (51)	0.79
Weight (kg)	80 ± 19	79 ± 20	0.80
SOFA score (without GCS)	2 (1-4)	3 (1-4)	0.89
Neurologic disorder^a^			0.08
Cerebrovascular disease	40 (53)	72 (66)	0.21
Acute hemorrhagic CVA	20 (27)	44 (40)	0.26
Acute thrombotic CVA	16 (21)	21 (19)	0.25
CVA, not classified	4 (5)	7 (6)	0.96
Other neuromuscular diseases	35 (47)	37 (34)	0.18
Peripheral neuropathy	12 (16)	7 (6)	0.14
Movement disorders	7 (9)	10 (9)	0.48
Multiple sclerosis	5 (7)	2 (2)	0.20
Other	11 (15)	18 (17)	0.14
Comorbidities			
Acute myocardial infarction	16 (21)	25 (23)	0.78
Heart failure	8 (11)	19 (17)	0.20
COPD	14 (19)	17 (16)	0.59
Diabetes mellitus	24 (32)	43 (39)	0.30
Obstructive sleep apnea	26 (35)	41 (38)	0.68
Endotracheal tube size			0.22
≤7.5 (*n *= 86)	31 (41)	55 (50)	
≥8.0 (*n *= 98)	44 (59)	54 (50)	
Intubated in emergency department	18 (24)	26 (24)	0.98
Duration from extubation to BSE (days)	1 (1-2)	2 (1-3.5)	0.45
Reintubation (before BSE)	3 (4)	12 (11)	0.07
Tracheostomy	6 (8)	30 (28)	<0.01
Ventilator days	4 (2-8)	10 (5-16)	<0.01
Mechanical ventilator >7 days	19 (25)	69 (63)	<0.01

Data presented as *n *(%), mean ± standard deviation or median (25th to 75th percentiles). BSE, bedside swallow evaluation; CVA, cerebrovascular accident; COPD, chronic obstructive pulmonary disease; GCS, Glasgow Coma Scale; SOFA, Sequential Organ Failure Assessment. ^a^When multiple neurologic disorders were present, the most dominant or limiting condition was used for this classification.

Dysphagia persisted at the time of discharge from the hospital for 82 of the 171 patients (48%) with any degree of dysphagia. Among the entire cohort, patients with moderate/severe dysphagia (66% (72/109)) were more likely than patients with mild dysphagia (16% (10/62)) to have persistent disease at the time of discharge (*P *<0.01). For those patients whose dysphagia had resolved, the median duration of dysphagia was 4 days (2.5 to 7.5 days). Among these patients who resolved, mild dysphagia (3 days (2 to 4 days)) lasted fewer days than moderate/severe dysphagia (7 days (5 to 14 days), *P *<0.01).

Univariate analyses evaluating for associations between the severity of dysphagia and patient outcomes are shown in Table 2. The presence of moderate/severe dysphagia was significantly associated with the number of hospital days after the initial BSE, no oral intake status, the surgical placement of a feeding tube, and discharge status (discharge to home as compared with either a long-term acute care, skilled nursing, or palliative care facility). The presence of moderate/severe dysphagia was not associated with pneumonia, reintubation, or hospital mortality. A multivariate analysis was fit for the duration of hospitalization after the initial BSE (Table 3). When adjusting for age, severity of illness, and tracheostomy, patients with moderate/severe dysphagia stayed in the hospital 4.32 days longer after their initial BSE (95% confidence interval = 3.04 to 5.60 days, *P *<0.01). No interaction terms were significant in this model.

**Table 2:** Associations between post-extubation dysphagia and poor patient outcomes

	Dysphagia severity		
		
Outcome	None/mild (*n *= 75)	Moderate/severe (*n *= 109)	*P *value
Pneumonia	6 (8)	11 (10)	0.63
Reintubation	4 (5)	11 (10)	0.24
In-hospital mortality	1 (1)	3 (3)	0.50
Pneumonia, reintubation, or death^a^	8 (11)	17 (16)	0.34
Persistent dysphagia at discharge	10 (13)	72 (66)	<0.01
Discharge to home	27 (36)	24 (22)	0.04
Kept no oral intake	7 (9)	90 (83)	<0.01
Surgical feeding tube	4 (5)	37 (34)	<0.01
Duration of hospitalization after BSE(days)	7.5 (5-12)	13 (9-20)	<0.01

Data presented as *n *(%) or median (25th to 75th percentiles). BSE, bedside swallow evaluation. ^a^Outcome data for pneumonia, reintubation and death are composite totals.

**Table 3:** Multivariate analysis^a ^of the effect on duration of hospitalization after initial bedside swallow evaluation

Term	β	95% confidence interval	*P *value
Intercept	17.51	12.40 to 22.62	<0.01
Age	0.04	-0.05 to 0.07	0.62
SOFA score	-0.08	-0.61 to 0.45	0.89
Tracheostomy	0.92	-0.73 to 2.57	0.58
Moderate/severe dysphagia	4.32	3.04 to 5.60	<0.01

SOFA, Sequential Organ Failure Assessment. ^a^Adjusted for age, severity of illness, and tracheostomy.

## Discussion

In a large cohort of critically ill, acute respiratory failure survivors with primary neurologic disorders, longer durations of mechanical ventilation are associated with the development of more severe post-extubation dysphagia in those patients without tracheostomy. Furthermore, dysphagia persists at the time of discharge in a large portion of these patients, as 66% (72/109) of those initially diagnosed with moderate and severe dysphagia had persistent disease at the time of discharge. Finally, moderate and severe dysphagia is independently associated with poorer patient outcomes, including longer hospital stays, reduced dietary intake, placement of feeding tubes, discharge to a nursing home, and a longer duration of hospitalization after the initial diagnosis of dysphagia.

While dysphagia occurs in 30 to 64% of nonintubated acute stroke survivors [12,19,20], little is known about the prevalence or cause of dysphagia in recentlyextubated critical illness survivors with primary neurologic disorders. Recentlyextubated patients in a neurocritical care unit can have dysphagia for multiple reasons [21]. First, irrespective of their acute respiratory failure, the initial neurologic insult may be the primary cause of impaired swallowing function at the time of extubation. A second mechanism is the laryngeal and pharyngeal damage that may be caused by the endotracheal tube. Thirdly, new dysfunction of both motor and sensory nerves can result from critical illness, impairing the complex choreography of swallowing. Next, an impaired sensorium, either related to ICU-acquired delirium, underling critical illness, or the effects of sedating medications, can result in aspiration, irrespective of the underlying neuromuscular function. Finally, the loss of the synchronous control of breathing and swallowing may result from underlying respiratory difficulty and tachypnea. While a study of this type is not able to determine the precise mechanisms present in our cohort of patients, further studies are necessary to determine the mechanisms acting in individual patients because this knowledge may lead to new targeted treatments aimed at reducing morbidity and mortality.

The independent association between the duration of mechanical ventilation and the severity of dysphagia supports the concept that endotracheal intubation may be associated with some degree of neuromotor and sensory damage, resulting in post-extubation dysphagia. Other studies showing an independent association between intubation duration and the severity of dysphagia support this association, although it is important to note that all these studies excluded patients with neurologic disorders [8,22-24]. The neuromotor and sensory mechanisms for swallowing dysfunction in newlyextubated patients in a neurocritical care unit deserve further investigation.

We report the novel finding that moderate/severe dysphagia on BSE is independently associated with a longer duration of hospitalization in a cohort of extubated critical illness survivors with neurologic impairment. This principle is partially supported by a recent review of the National Hospital Discharge Survey, which revealed a significant association between the presence of dysphagia and hospital length of stay and mortality in predominantly nonintubated patients [25]. The annual national cost of aspiration and pneumonia in patients recovering from an acute cerebrovascular disorder is estimated to be $459 million [26]. Furthermore, the daily cost for hospitalized patients was recently estimated to be $1,153 for a noncritical care bed and $3,518 for a critical care bed, representing a 30% increase from the years 2000 to 2005 [27]. These figures highlight the need to further investigate the role for dysphagia screening and treatment in recentlyextubatedneurocritical care patients.

Our study has several limitations. Sixty-seven percent of patients who survived to be extubated in our study did not undergo a BSE. We could only study dysphagia in those patients who had received a BSE. Second, data collection was limited by a few inconsistently charted variables. For example, we were unable to obtain the following: Glasgow Coma Score data to include in the SOFA score; a reliable marker of sedation at the time of swallow evaluation; and height data to calculate both body mass index and height/endotracheal tube diameter [28]. Third, we were unable to adjust for preadmission functional state or the presence of known, pre-existing dysphagia. Therefore, it is not clear from this study whether patients had dysphagia as a result of their intubation or had dysphagia that happened to be diagnosed following intubation. Because we were unable to control for preadmission functional state or the presence of pre-existing dysphasia, it is possible that our results are confounded by these conditions.

Finally, a significant limitation in both our study and this medical field is the lack of a widelyperformed and validated diagnostic test for dysphagia and aspiration. The BSE interpretation is based on the judgment of the speech-language pathologist, and the accuracy of the BSE in recentlyextubated patients has not been proven, which may have led to misclassification in our study. We feel that our conclusions are still important, however, because misclassification due to our subjective speech-language pathologist assessments would most probably bias our results towards the null hypothesis. The BSE misses silent aspiration or aspiration that is not associated with external signs, such as coughing or choking. If all of the misclassification occurred through the poor detection of silent aspiration, it is also possible that those patients with silent aspiration could have had poorer outcomes because they were given more liberal dietary treatment. Leder and colleagues evaluated 20 consecutive extubated trauma patients with a fiberoptic endoscopic evaluation of swallowing, widelyregarded to be the gold standard test for post-extubation dysphagia [29-31], and found that aspiration was present in 45% of all subjects, and was silent just under one-half of the time [32]. Ajemian and colleagues also reported a silent aspiration frequency of 25% in a mixed population of medical and surgical patients [33]. In both of these studies, in which fiberoptic endoscopic evaluation of swallowing was used to guide nutritional decisions, aspiration was not associated with patient mortality. However, the clinical benefit of a fiberoptic endoscopic evaluation of swallowing approach to nutrition on other outcomes in recentlyextubated patients with neurologic disorders has not been explored in larger prospective studies.

We can hypothesize as to the reasons for our findings. First, the duration of mechanical ventilation is probably positively associated with one or many of the mechanisms of swallowing dysfunction that we described above. Most logically, it would seem that patients who are intubated longer have a higher likelihood of developing oral, pharyngeal, and laryngeal injuries. Similarly, these patients also may be at higher risk for critical illness polyneuromyopathy, which may affect the bulbar, as well as the peripheral, muscles and nerves. With regard to the duration of hospitalization, patients with impaired nutrition and/or the presence of feeding tubes probably stay longer, on average, in the hospital. Whether this is due to patient, family, and/or physician preferences or other factors, and whether the swallowing dysfunction represents a modifiable risk factor for longer hospital stays, will need to be confirmed in a larger prospective study.

## Conclusion

In summary, the development of post-extubation dysphagia is associated with poorer outcomes in survivors of acute respiratory failure who required mechanical ventilation and had neuromuscular or cerebrovascular disease. Additionally, longer durations of mechanical ventilation are associated with the development of post-extubation dysphagia in these patients. Investigating the exact mechanisms of post-extubation dysphagia in this patient population, defining those at highest risk for the disease, and exploring novel treatment options for this disorder could potentially decrease morbidity for a significant percentage of patients recovering from acute respiratory failure.

## Key messages

• Post-extubation dysphagia, or swallowing dysfunction occurring after cessation of mechanical ventilation and the removal of endotracheal tubes, is potentially common in a large population of medical and surgical ICU patients with either acute or pre-existing neurologic disease.

• This study suggests an independent association between post-extubation dysphagia in this population and poor patient outcomes, including hospital length of stay.

• This study shows that longer durations of mechanical ventilation are associated with the development of post-extubation dysphagia in patients with either acute or pre-existing neurologic disease.

• Post-extubation dysphagia persists at the time of discharge in a large portion of patients (48%, 82/171).

• Post-extubation dysphagia is an under-recognized and potentially costly form of impairment in survivors of critical illness with neurologic impairment. Further research is necessary to separate the different etiologies and the precise neuromuscular mechanisms for this disorder.

## Abbreviations

BSE: bedside swallow evaluation; CVA: cerebrovascular accident; SOFA: Sequential Organ Failure Assessment.

## Competing interests

The authors declare that they have no competing interests.

## Authors' contributions

MMa, CJK, and MMo conceived of the study and contributed to the data analysis and manuscript preparation. MMa and CJK also contributed to the data collection. TW participated in the study design and also contributed to the data collection. BJC, ABB, and ELB contributed to the study design, analysis, and manuscript preparation. AW contributed to the statistical analysis and manuscript preparation. All authors read and approved the final manuscript.

## Authors' information

MMa, CJK, BJC, ABB, ELB, and MMo are members of the Division of Pulmonary Sciences and Critical Care Medicine, University of Colorado Denver. TW is the Director of Rehabilitation Services at the University of Colorado Hospital. AW is a member of the Division of Biostatistics and Bioinformatics, National Jewish Health.
